# Have Elbow Arthroscopy Hospitalizations Decreased over the Years? An Epidemiological Italian Study from 2001 to 2016

**DOI:** 10.3390/ijerph20043638

**Published:** 2023-02-18

**Authors:** Umile Giuseppe Longo, Rocco Papalia, Sergio De Salvatore, Valentina Piccioni, Alessandro Tancioni, Ilaria Piergentili, Vincenzo Denaro

**Affiliations:** 1 Research Unit of Orthopaedic and Trauma Surgery, Fondazione Policlinico Universitario Campus Bio-Medico, Via Alvaro del Portillo, 00128 Roma, Italy; 2 Research Unit of Orthopaedic and Trauma Surgery, Department of Medicine and Surgery, Università Campus Bio-Medico di Roma, Via Alvaro del Portillo, 21, 00128 Roma, Italy

**Keywords:** elbow arthroscopy, surgery, non-invasive, demographic trends, adult, incidence, fracture, instability

## Abstract

This study describes the trends of elbow arthroscopy in Italy and other countries in order to evaluate the yearly rates of EA. Its purpose is for future epidemiological studies to be able to compare their data between countries in order to understand the reasons for the increasing and decreasing trends. Data for this study were obtained from National Hospital Discharge records (SDO) at the Italian Ministry of Health (INHS). Data regarding sex, age, region of residence, region of surgery, length of hospitalization, and procedure codes were included. In total, 2414 elbow arthroscopies were performed in Italy from 2001 to 2016 in the adult population. The highest number of procedures was found in the 40–44 and 45–49 years age groups. Males represented the majority of patients undergoing EA both in total and over the years. An increase from 2001 to 2010 and a decrease from 2010 to 2016 were reported in the present analysis. According to other studies, males of 40–44 and 45–49 years age groups represent the most treated patients. Further epidemiological studies would provide data that could be compared between countries, reaching a general consensus on the best indications for this procedure.

## 1. Introduction

Elbow arthroscopy (EA) is a surgical procedure that is utilized to treat various complex pathological conditions affecting the elbow joint, such as degenerative arthritis, synovitis, extrinsic rigidity, lateral epicondylitis, radial head resection, posterolateral rotatory instability, fractures of the capitellum, coronoid, and radial head [1,2]. However, despite its benefits, such as the reduced risk of infection and minimal scarring compared to open surgery, the arthroscopy technique comes with its limitations and potential hazards [3,4].

The surgeon must understand the intricacies of EA and how it fits into the surgical practice in order to make informed decisions that are in the best interest of the patient [3,4]. This procedure is technically challenging and requires extensive training and experience to avoid significant complications [1]. The current literature reports several potential complications, including peripheral nerve injury, joint stiffness, thromboembolic complications, and septic arthritis [3,5,6,7]. In addition, the risk of neurological injury during EA is higher than in other joints due to the proximity of several neurovascular structures to the established portals [4,7,8].

Despite the increasing number of indications for EA, it is crucial to be aware of the potential complications and consequences of this surgical procedure [4,9]. Although complications with EA occur in 14% of cases, serious consequences are rare and occur in only 5% of cases [3]. With proper judgment and technique, EA can be a valuable tool for correcting a range of elbow joint lesions. The success of the procedure ultimately depends on thorough preoperative planning, including a comprehensive history and physical examination, appropriate imaging studies, and clinical judgment [7]. A fundamental understanding of the anatomy of the elbow is necessary for comprehending the relationship between neurovascular structures and portals. To maximize the success rate of EA, the focus must be placed on proper work-up, anesthesia, patient positioning, portal placement, and appropriate instruments [10]. 

There is limited epidemiological data available in the literature on the prevalence of EA, with only a few studies reporting nationwide incidences in Europe and Southern California [11,12]. Therefore, the aim of this study is to report the demographic trends of hospitalizations for EA from 2001 to 2016, based on national hospitalization records, to contribute to the international comparison of outcomes associated with this procedure.

## 2. Materials and Methods

The National Hospital Discharge records (SDO) for EA from 2001 to 2016 were used. This registry was provided by the Italian Ministry of Health from both private and public Italian hospitals [13,14,15,16]. The anonymous data included the age, sex, region of residence, region of surgery, length of hospitalization stay of the patients, and the diagnosis and procedure codes. The primary procedure code by the International Classification of Diseases, Ninth Revision, Clinical Modification (ICD-9-CM) of EA was 80.22. Only patients over 15 years old were included in the study. The adult annual population size was obtained from the National Institute for Statistics (ISTAT).

### Statistics

The IBM SPSS Statistics for Windows, Version 26.0. (Armonk, NY, USA: IBM Corp) and Microsoft Excel (2019) was used. Frequencies and percentages were calculated for categorical data, and for continuous data, averages and standard deviations were used. The incidence was calculated as the ratio between the number of cases and the size of the adult population, with reference to 100,000 inhabitants (cases/population × 100,000).

## 3. Results

### 3.1. Demographics

**Figure 1:**
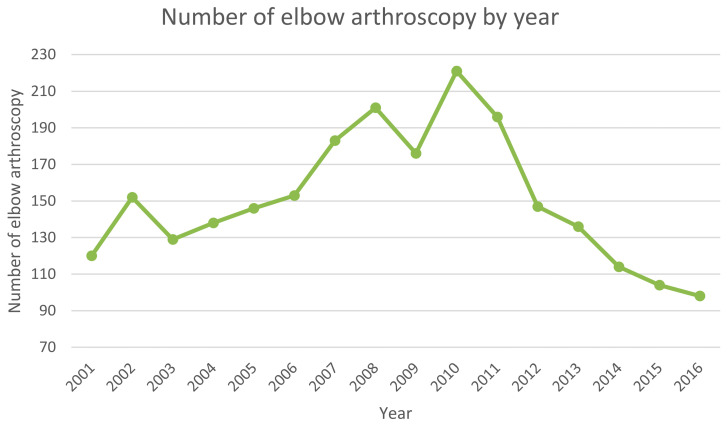
Number of elbow arthroscopy by year.

In total, 2414 EAs were executed between 2001 and 2016 in the adult population. The number of EAs showed an increase from 2001 to 2010 and a decrease from 2010 to 2016 (Figure 1).

**Figure 2:**
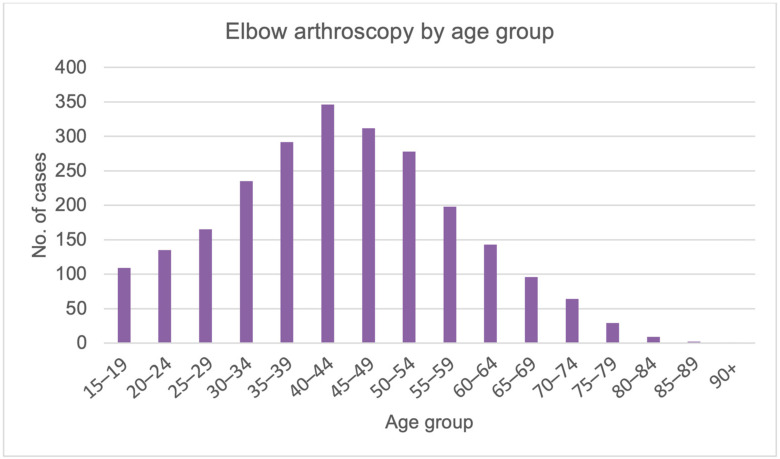
Elbow arthroscopy by age group.

The cumulative period of incidence was 0.3 EA for every 100,000 Italian inhabitants over 15 years old. Overall, the highest number of procedures was found in the 40–44 (14.3%) and 45–49 (12.9%) year age groups (Figure 2).

**Figure 3:**
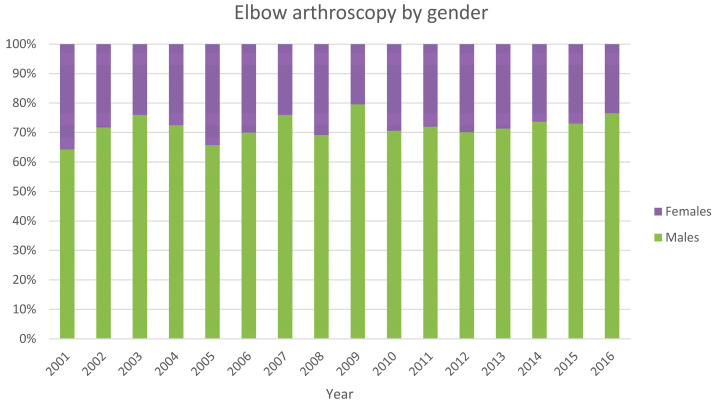
Elbow arthroscopy by gender.

The male/female ratio was 2.6 (from 1.8 in 2001 to 3.3 in 2016). Males represented the majority of patients who underwent EA procedures, both in total and over the years (female 28% and male 72%) (Figure 3).

**Figure 4:**
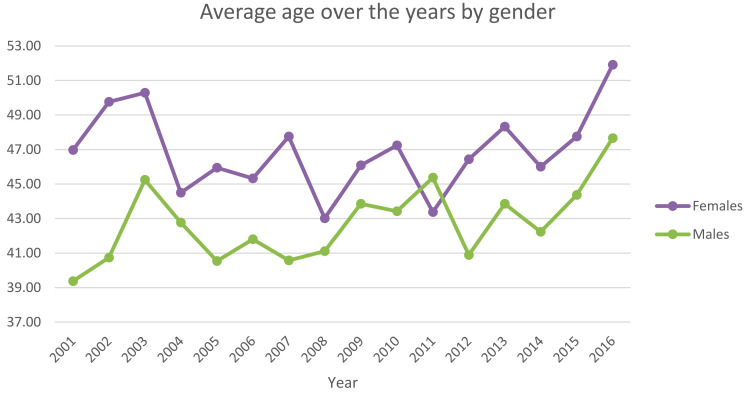
Average age over the years by gender.

The average age of patients was 43.8 ± 14.3 (46.6 ± 15 years for females and 42.7 ± 13.8 years for males). Only in 2011 was the average age of males higher than females (Figure 4).

### 3.2. Length of the Hospitalization

**Figure 5:**
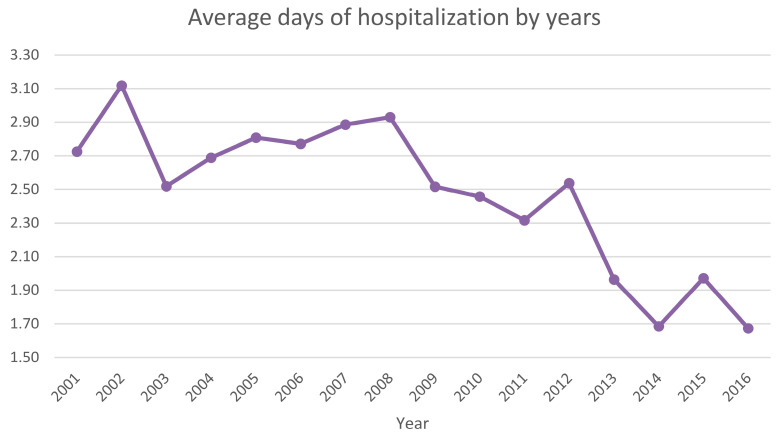
Average days of hospitalization by years.

The average days of hospital stay were 2.5 ± 2.9 days, from a minimum of 1 to a maximum of 79 days (females 2.6 ± 2.7 days and males 2.5 ± 2.9 days). The decreasing trend of the mean number of days of hospitalization is shown in Figure 5.

**Figure 6:**
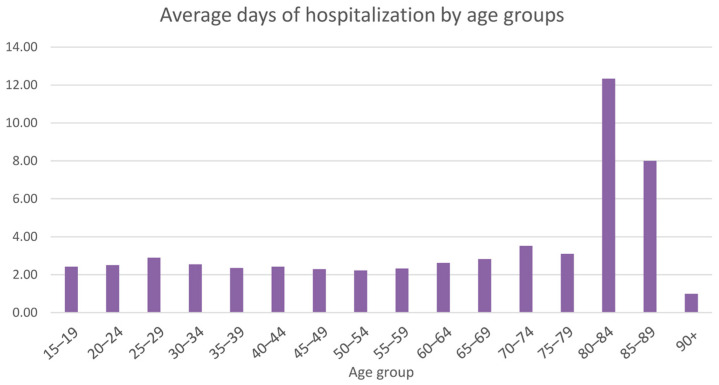
Average days of hospitalization by age groups.

Older patients had more days of hospitalization on average (Figure 6).

### 3.3. Main Primary Diagnoses

During the 16-year study period, the main primary diagnoses were stiffness of the joint, not elsewhere classified, in the upper arm (ICD code: 719.52; 28.6%), lateral epicondylitis (ICD code: 726.32; 7.9%), and contracture of the joint in the upper arm (ICD code: 718.42; 6.3%).

### 3.4. Economic Impact

**Figure 7:**
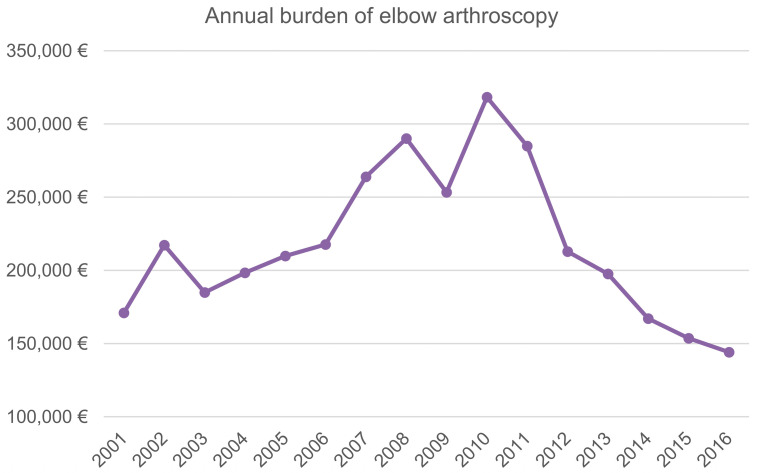
Annual burden of elbow arthroscopy.

The actual mean Italian hospital reimbursement ranges from EUR 1361 (more than one-day stay, with an increment of EUR 7 for every extra day of hospitalization) to EUR 1512 (one-day stay procedure) for each EA hospital admission. Overall, from 2001 to 2016, a total cost of EUR 3484,074 has been assessed. The annual mean cost was about EUR 217,754 ± EUR 51,311 with a range from EUR 144,108 in 2016 to EUR 318,286 in 2010 for EA procedures in Italy (Figure 7).

## 4. Discussion

In Italy, from 2001 to 2016, the number of EA per year decreased as the mean length of stay decreased. In specific, the number of EA hospitalizations increased from 2001 to 2010 and decreased from 2010 to 2016. During the 16-year study period, male patients received the majority of elbow arthroscopic surgeries with contracture of the joint, lateral epicondylitis, and stiffness of the joint as the main primary diagnosis.

A study conducted at the University of Southern California, by Leong et al., reported a slight increase in arthroscopic elbow procedures from 2007 to 2011 as in Italy [12]. However, according to Leong and colleagues, this increase is not connected to any specific reason [12]. Male patients accounted for 71% of American patients undergoing these procedures. Much like in Italy, the percentage of males is higher than that of females. The data regarding gender and age distribution of the two studies is very similar. Indeed, EA was common in male patients in the 40 to 59-year-old range. This result is in line with the fact that EA procedures are frequently observed in middle-aged patients with degenerative elbow disease. In Leong et al.’s study, the incidence of EA procedures nationwide did not differ significantly by area, while in Italy, 68.1% of EA cases were recognized in the northern regions [12].

Karelsonb et al., in a Finnish study, observed an increase in EA procedures from 1997 to 2010, but then, the incidence of these arthroscopic operations peaked in the years 2012 and 2014 [11]. These results match the findings of the present study. According to Karelsonb and colleagues, there are no clinical trial reports to explain why the rates of EA decreased [11]. However, one possible reason for the decreasing rates of arthroscopic procedures can be related to organizational factors based on the increased concern for cost-effective healthcare interventions [11].

To our knowledge, no article has been published regarding the costs of EA. In order to be able to make comparisons with future studies, the costs of EA in Italy from 2001 to 2016 were estimated. During the 16 years of study, a total cost of EUR 3,484,074 has been assessed. The annual mean cost was about EUR 217,754 ± EUR 51,311 with a range from EUR 144,108 in 2016 to EUR 318,286 in 2010. In an expanding cost-conscious healthcare setting, the economic analysis of surgical procedures is mandatory, and further cost-analysis studies are required [17]. 

Arthroscopies are now a standard procedure in modern orthopedic surgery [7,18]. Increased knowledge and understanding of the anatomy of the elbow and EA procedures developed in recent years; thus, the indications for EA have been expanded by surgeons [4]. The elbow has a high degree of natural stability supported by osseous and soft-tissue limitations; nonetheless, instability of the elbow joint occurs when significant lesions of these sustaining structures take place. Regardless of the advancements, the EA surgical procedure can be difficult, and the rate of persistent instability, post-traumatic arthritis, stiffness, and discomfort can still be significant, especially in the most difficult cases. An arthroscopic evaluation may be recommended in some cases of acute or chronic instability, allowing for the assessment of the condition’s severity and the implementation of treatment [19]. EA is especially effective when the physical examination and radiographic findings are unclear [20,21]. Indeed, arthroscopy represents an additional diagnostic tool to determine the degree of elbow joint instability. However, it should be carried out when subsequent surgical intervention is possibly needed. Because of this, the history, examination, and other available resources should be taken into account while selecting and evaluating diagnostic techniques [19]. It is a less invasive tool to confirm the diagnosis of an infection, to debride infected tissue, to remove loose bodies and osteophytes, and to treat lateral epicondylitis [6]. With reference to lateral epicondylitis, two clinical tests have been developed: Supination and Antero-Lateral pain Test (SALT) and Posterior Elbow Pain by Palpation–Extension of the Radio-capitellar joint (PEPPER). These two new tests can provide a reasonable tool to assess lateral elbow pain to help in diagnosis before arthroscopic surgery [22]. Nowadays, the range of indications has increased to include treatment for more difficult pathologies including radial head resection, the management of intraarticular fractures, total synovectomy, and ligament repair [23]. The use of arthroscopy procedures will increase as they become more standardized and common, lowering the risk of stiffness and speeding up recovery [19].

EA arthroscopy is performed in a supine position. It involves the use of two arthroscopes at different angles to provide a comprehensive and unobstructed view of the operative field. The success of this procedure relies on a thorough preoperative work-up, which helps to minimize the risk of perioperative complications and avoid unnecessary surgery [10,24].

However, the surgery can become complicated due to various factors, such as prior trauma or surgery, rheumatoid arthritis, a subluxating ulnar nerve, or congenital malformations of the elbow. Therefore, it is imperative to properly identify the anatomic landmarks and portal sites before incision marking. This helps to avoid any potential nerve injuries or other complications during the procedure. Once the patient is positioned, fluid distension of the elbow joint is performed to move away the neurovascular structures and establish proximal portals to the joint. The surgeon must maintain proper visualization of the instrument tips in proximity to the nervous structures through the pronation and flexion of the elbow. The handling of arthroscopic instruments is highly dependent on the surgeon’s experience and expertise. As such, safe and effective EA procedures require the expertise of a competent elbow arthroscopist. Young surgeons who are new to this field should undergo thorough training including coaching from an experienced elbow arthroscopist, computer-simulated courses, and hands-on cadaveric courses to ensure they are adequately prepared to perform this procedure [10,24].

Compared to patients treated with open techniques, EA patients benefit from many advantages in the hands of an experienced surgeon [4,20]. Since patients undergoing EA benefit from enhanced elbow joint visualization, the possibility to look for coexisting intra-articular pathologies, and minimal soft tissue injury with no negative impact on clinical outcomes, the range of indications for EA is expanding [5,20]. Arthroscopy of the elbow has become effective in the treatment of elbow trauma over the last decade [1]. In the future, the integration of arthroscopy with new technologies (augmented reality and artificial intelligence) could improve the accuracy and effectiveness of this procedure [25]. In fracture treatment, arthroscopy has been used in the displacement of the radial head, coronoid and capitellum fractures in adults, and displaced radial neck and lateral humeral condyle fractures in children with good results [1,26]. As a matter of fact, even though in pediatric and adolescent patients the relationship between structures in the elbow joint is closer, EA is not riskier than it is in adults [27].

EA allows quite rapid rehabilitation and requires a few days of hospitalization; this means an earlier return to normal life so to work and daily practice [21,28]. On the other hand, open elbow surgery will require much more time. The patient undergoing the EA procedure will be able to return to daily activities in about 6 weeks, will go back to work in 12 weeks more or less (depending on the job), and will be able to play sports again in 4 to 6 months [21]. The EA procedure provided great results for elite athletes subject to constant traumatic and non-traumatic stress. In particular, given the structural and mechanical complexity of the elbow joint, it is commonly subject to the development of stiffness and rigidity [2]. The surgery improved athletes’ quality of life, reducing elbow stiffness and increasing the range of motion of the elbow joint [29].

EA is a technically difficult procedure that demands substantial hands-on training and supervised practice to master [6]. Arthroscopy is a technically challenging procedure with numerous possible complications due to the close proximity of neurovascular structures and the small joint spaces [5]. During an EA procedure, there is the possibility to make iatrogenic damage to the radial and ulnar nerve due to the presence of many important major neurovascular structures very near to the operative portals [4,7]. According to Jessica Intravia and colleagues, nerve injury rates are 0.5–10.4% with only 2% neurological sequelae. Heterotopic ossification may also result from EA, and it is reported in 2.5% of cases [30]. Moreover, Gregory N. Nelson et al., in a study of 417 procedures, reported a rate of superficial and deep infections of 6.7% and 2.2%, respectively [3]. Indeed, postoperative infection is a major side effect of EA, as reported by Jessica Intravia et al. (2% for superficial infections and 0.5% for deep infections [30]). Therefore, even if septic arthritis after EA is infrequent, its effects can be devastating to the patient and often leads to a prolonged course of antibiotics and a decline in joint function [5,7,8,31].

The present study may help to understand the yearly trends of EA taking into consideration the economic analysis of this procedure in Italy. However, due to the lack of cost-effectiveness studies worldwide, it was not possible to compare it with other countries. To our knowledge, this article is the first national economic analysis on EA. Since the literature lacks epidemiological studies on EA, this evaluation could be used to compare and understand the rates of EA procedures around the world. Moreover, the strength of this epidemiological study is the use of a validated nationwide database describing the use of EA in Italy; thus, changes in prevalence over time mirror the general opinion on this surgical procedure. 

### Limitations

There are some limitations to this study. The International Classification of Diseases 9 (ICD-9) was used for all the procedures reported. However, some hospitals employ different codes to record EA. The heterogeneity of codification among different hospitals could lead to an underestimation of the results. Patient-specific information, surgical report details, post-surgery rehabilitation procedures, and outcome data were available with anonymous ID numbers for reasons of privacy; therefore, the complication rate was not taken into account in this investigation. The data utilized in our study were obtained directly from the Italian Ministry of Health. All public and private hospitals in Italy are required to periodically submit National Hospital Discharge records (SDO) to the Ministry. However, it is worth noting that the Ministry does not make these records publicly available on an annual basis. As a result, the data used in our study represent the most recent records that are currently available within the limitations of the Italian health information system. Lastly, this is an epidemiological study; therefore, it is not possible to define the reasons for the change in the trend in this procedure over the years. However, the lack of a reason for the change in trends is also reported by other authors [11,12].

## 5. Conclusions

EA has developed significantly and the indications for arthroscopy have expanded as methods and technology have advanced. Although arthroscopy has several benefits over open surgery, the structure of the elbow joint and the anatomical relationships around it makes EA surgery challenging. To avoid serious problems, arthroscopy is a technically difficult procedure that calls for substantial training and experience. Two peak modalities were recorded in EA hospitalizations in Italy. In specific, an increase from 2001 to 2010 and a decrease from 2010 to 2016 were reported in the present analysis. According to other studies, males of the 40–44 and 45–49 years age groups represent the most treated patients. The lack of international data and registers on EA makes it challenging to provide international guidelines on EA. Further epidemiological studies would provide data that could be compared between countries, reaching a general consensus on the best indications for this procedure.

## Data Availability

The data presented in this study are available on request from the corresponding author.
